# Supplementary material for: Homologous and Heterologous Anti-COVID-19 Vaccination Does Not Induce New-Onset Formation of Autoantibodies Typically Accompanying Lupus Erythematodes, Rheumatoid Arthritis, Celiac Disease and Antiphospholipid Syndrome
Source: Vaccines (Basel). 2022 Feb 18;10(2):333. doi: 10.3390/vaccines10020333 (PMC8880348; doi:10.3390/vaccines10020333)
Supplement: Supplementary file 1 [file vaccines-10-00333-s001.zip › vaccines-1577476-supplementary.pdf]

vaccines

MDPI

Supplementary figures

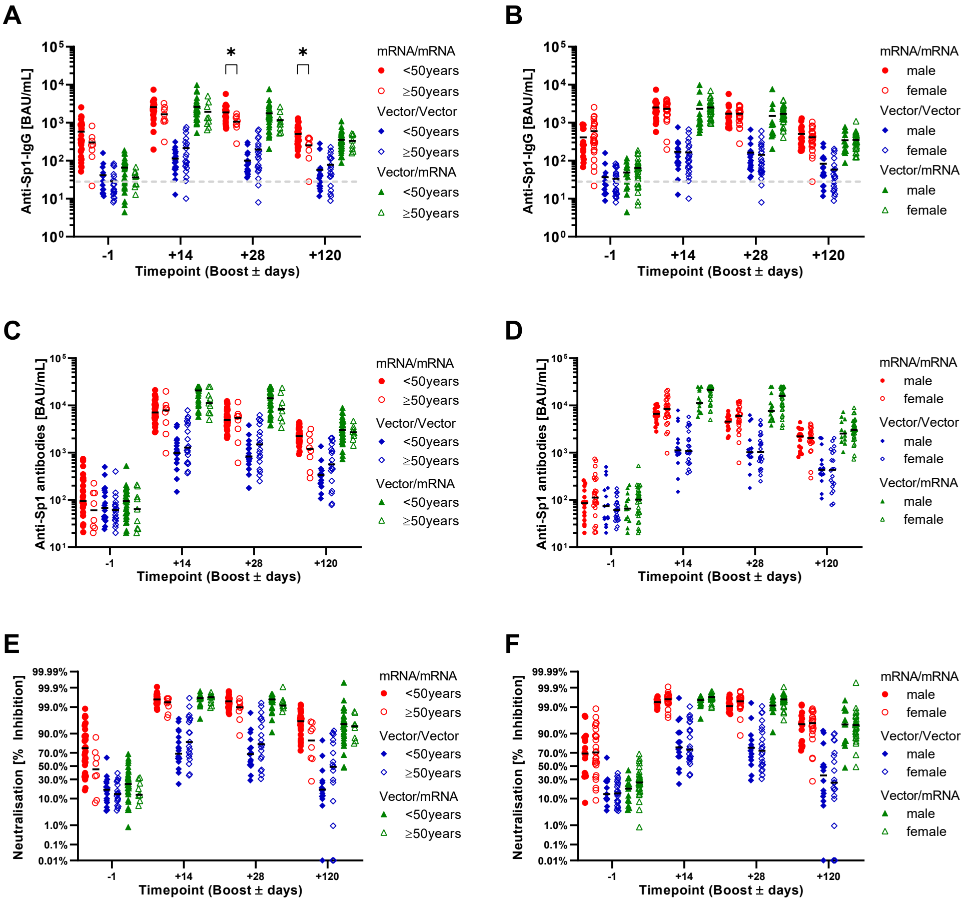

Figure S1: Analysis of anti-SARS-CoV-2-Sp1 antibodies in the different groups (red – mRNA/mRNA, blue – Vector/Vector, green mRNA/Vector) with respect to age and gender. (A, C, and E) Anti-SARS-CoV-2-Sp1-IgG (A), anti-SARS-CoV-2-Sp1 antibodies (C) and neutralizing antibodies (E) in individuals <50 years (filled symbols) and ≥50 years (open symbols). **(B, D, and F)** Anti-SARS-CoV-2-Sp1-IgG (B), anti-SARS-CoV-2-Sp1 antibodies (D) and neutralizing antibodies (F) in males (filled symbols) and females (open symbols). Statistical analyses by Mixed-effects analysis with Tukey's multiple comparison test **(A)**. * $p<0.05$

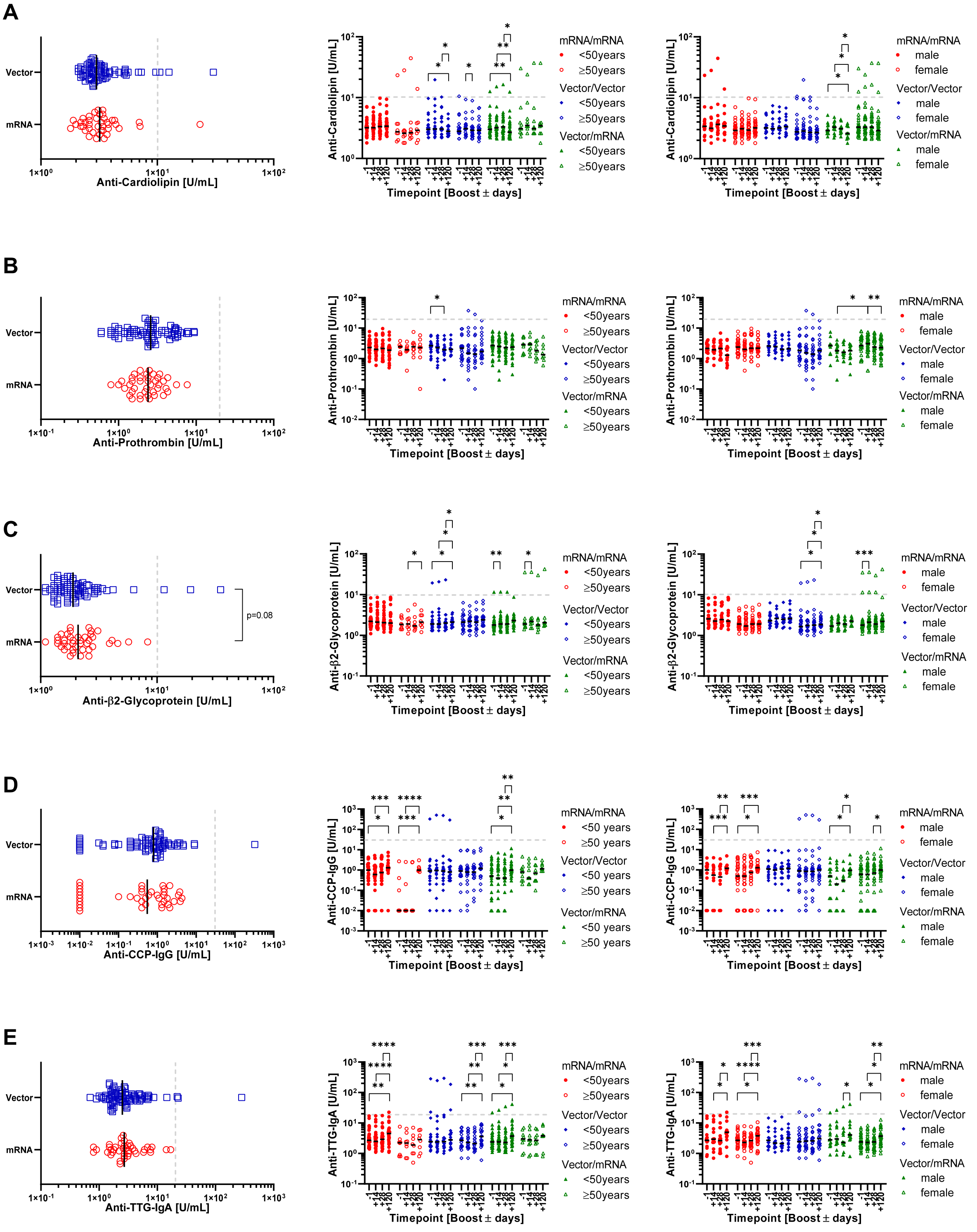

Figure S2: Analysis of autoantibodies based on prime vaccinations in serum samples 1 day before boost (blue – vector prime, red – mRNA prime) and in the different groups (red – mRNA/mRNA, blue – Vector/Vector, green mRNA/Vector) with respect to age and gender. (A) Anti-Cardiolipin antibodies: left, autoantibody levels before boost based on prime vaccinations; middle, analysis in individuals <50 years (filled symbols) and ≥50 years (open symbols) based on the study groups; right, analysis in males (filled symbols) and females (open symbols) based on the study groups. **(B)** Anti-Prothrombin antibodies: left, autoantibody levels before boost based on prime vaccinations; middle, analysis in

individuals <50 years (filled symbols) and ≥50 years (open symbols) based on the study groups; right, analysis in males (filled symbols) and females (open symbols) based on the study groups. **(C)** Anti-β2-Glycoprotein antibodies: left, autoantibody levels before boost based on prime vaccinations; middle, analysis in individuals <50 years (filled symbols) and ≥50 years (open symbols) based on the study groups; right, analysis in males (filled symbols) and females (open symbols) based on the study groups. **(D)** Anti-CCP antibodies: left, autoantibody levels before boost based on prime vaccinations; middle, analysis in individuals <50 years (filled symbols) and ≥50 years (open symbols) based on the study groups; right, analysis in males (filled symbols) and females (open symbols) based on the study groups. **(E)** Anti-TTG antibodies: left, autoantibody levels before boost based on prime vaccinations; middle, analysis in individuals <50 years (filled symbols) and ≥50 years (open symbols) based on the study groups; right, analysis in males (filled symbols) and females (open symbols) based on the study groups. Statistical analyses by Mixed-effects analysis with Tukey's multiple comparison test within and between groups **(A-C)**. * p<0.05, ** p<0.01, *** p<0,001, **** p<0,001

Table 1. Number of samples analyzed with the different methods at the different timepoints.

	**mRNA/mRNA**				**Vector/Vector**				**Vector/mRNA**			
**timepoint**	**-1**	**14**	**28**	**120**	**-1**	**14**	**28**	**120**	**-1**	**14**	**28**	**120**
**anti-SARS-CoV-2-Sp1-IgG**	41	41	40	39	37	37	37	34	42	42	42	41
**anti-SARS-CoV-2-Sp1 antibodies**	41	41	40	38	37	37	37	34	42	41	41	41
**neutralizing antibodies**	41	41	40	38	37	37	37	34	42	42	42	41
**IFN-γ release assay**				39				33				41
**anti-Prothrombin**	41	41	40	39	37	37	37	34	42	42	42	41
**anti-Cardiolipin**	41	41	40	39	37	37	37	34	42	42	42	41
**anti-β2-Glycoprotein**	41	41	40	39	37	37	37	34	42	42	42	41
**anti-CCP**	41	41	40	39	37	37	37	34	42	42	42	41
**anti-TTG-IgA**	41	41	40	39	37	37	37	34	42	42	42	41
**ANA**	41	41	40	39	37	37	37	34	42	42	42	41
